# Prognostic impact of peripheral blood *WT1* mRNA dynamics in patients with acute myeloid leukemia treated with venetoclax combination therapy

**DOI:** 10.1007/s10147-024-02480-9

**Published:** 2024-02-09

**Authors:** Honami Sato, Takahiro Kobayashi, Yoshihiro Kameoka, Kazuaki Teshima, Atsushi Watanabe, Masahiro Yamada, Takaya Yamashita, Shinsuke Noguchi, Yoshihiro Michisita, Naohito Fujishima, Jun Kuroki, Naoto Takahashi

**Affiliations:** 1 Department of Hematology, Nephrology, and Rheumatology, Akita University Graduate School of Medicine, 1-1-1 Hondo, Akita, 010-8543 Japan; 2 Department of Hematology, Hiraka General Hospital, Yokote, Japan; 3 Department of Hematology, Nephrology and Rheumatology, Omagari Kousei Medical Center, Daisen, Japan; 4 Department of Hematology, Akita City Hospital, Akita, Japan; 5 Department of Hematology, Akita Red Cross Hospital, Akita, Japan; 6 Department of Hematology, Akita Kousei Medical Center, Akita, Japan; 7 Department of Hematology, Nephrology and Rheumatology, Nohsiro Kousei Medical Center, Noshiro, Japan; 8 Department of Internal Medicine, Yuri Kumiai General Hospital, Yurihonjo, Japan

**Keywords:** Acute myeloid leukemia, Venetoclax, Azacitidine, Measurable residual disease, *WT1* mRNA, Predictive factor

## Abstract

**Background:**

*Wilms' tumor gene 1* (*WT1*) mRNA quantification is a useful marker of measurable residual disease in acute myeloid leukemia (AML). However, whether monitoring the *WT1* mRNA levels may predict the outcome of venetoclax (VEN) combination therapy in AML is not reported. This study aims to elucidate whether *WT1* mRNA dynamics could predict long-term prognosis.

**Methods:**

33 patients with untreated or relapsed/refractory AML evaluated for peripheral blood WT1 dynamics in VEN combination therapy were analyzed.

**Results:**

The median age was 73 years (range 39–87). Azacitidine was combined with VEN in 91% of patients. Overall, the median overall survival (OS) was 334 days (95% CI 210–482), and the complete remission (CR) plus CR with incomplete hematologic recovery rate was 59%. A 1-log reduction of *WT1* mRNA values by the end of cycle 2 of treatment was associated with significantly better OS and event-free survival (EFS) (median OS 482 days vs. 237 days, p = 0.049; median EFS 270 days vs. 125 days, p = 0.02). The negativity of post-treatment *WT1* mRNA value during the treatment was associated with significantly better OS and EFS (median OS 482 days vs. 256 days, p = 0.02; median EFS not reached vs. 150 days, p = 0.005). Multivariate analysis confirmed the significance of these two parameters as strong EFS predictors (HR 0.26, p = 0.024 and HR 0.15, p = 0.013, respectively). The increase in *WT1* mRNA values was correlated with relapse.

**Conclusion:**

This study demonstrates that *WT1* mRNA dynamics can be a useful marker for assessing long-term prognosis of VEN combination therapy for AML.

## Introduction

Elderly patients with acute myeloid leukemia (AML) ineligible for intensive chemotherapy have a poor prognosis [1–3]. However, the combination of venetoclax (VEN), a selective BCL-2 inhibitor, and azacitidine (AZA) or low-dose cytarabine (LDAC) is expected to improve the prognosis in these patients [4, 5].

Besides the cytogenetic abnormality at the diagnosis of AML [6], the degree of post-treatment measurable residual disease (MRD) is an important prognostic factor [7, 8]. The results of the phase III VIALE-A trial showed that among patients who achieved a complete remission (CR) plus CR with incomplete hematologic recovery (CRi) after VEN/AZA treatment, those who achieved multiparameter flow cytometry (MFC)-MRD < 10^–3^ had significantly longer overall survival (OS) (hazard ratio (HR) 0.285) and event-free survival (EFS), indicating that MRD is a strong predictor of OS [9]. Thus, longitudinal evaluation of MRD is necessary to predict the long-term outcome of AML patients treated with VEN combination therapy. However, MRD evaluation in MFC requires periodic bone marrow examination and given that no validated assay has been established, it cannot be performed in routine clinical practice. Therefore, establishing simpler universal MRD markers is desired in AML treated with VEN combination therapy.

The Wilms' tumor gene 1 (*WT1*) was identified as a tumor suppressor gene for pediatric kidney Wilms' tumor [10]. *WT1* mRNA is overexpressed at least 80% of patients with AML and can be universally used as an MRD marker, even in patients with AML who are not eligible for leukemia-specific polymerase chain reaction (PCR) assays (e.g., for NPM1, PML-RARA, or CBF AML) [11, 12]. Several studies have shown that high PB *WT1* mRNA values after treatment of AML are associated with relapse and poor prognosis [13–15]. However, the usefulness of PB *WT1* mRNA quantification as a predictor of long-term prognosis in VEN combination therapy for AML has not been reported. In this study, we evaluated the usefulness of PB *WT1* mRNA dynamics in patients with untreated or relapsed/refractory AML treated by VEN combination therapy. We found that post-treatment PB *WT1* mRNA values are associated with long-term prognosis.

## Patients and methods

### Study design

The multicenter prospective observational study was conducted in Akita prefecture, Japan. This study enrolled patients with AML who were judged ineligible for intensive chemotherapy by the attending physician and treated by VEN combination therapy. It was conducted in accordance with the 1964 Helsinki Declaration and approved by the Ethics Committee of Akita University (August 6, 2021/No. 2696). Written informed consent was obtained from all participants before enrollment.

### Patients

In this paper, we analyzed untreated or relapsed/refractory AML patients who could be evaluated for changes of PB *WT1* mRNA in the multicenter prospective observational study.

### Dosing regimen

VEN combined either with AZA (75 mg/m^2^ subcutaneously for 7 consecutive days, repeated after 28 days each) or LDAC (20 mg/m^2^ subcutaneously for 10 consecutive days, repeated after 28 days) was administered orally once daily using a 28-day cycle of therapy. The VEN dose ramp-up was conducted over 3 to 4 days in accordance with the package insert to mitigate the risk of tumor lysis syndrome. The dose adjustment of VEN was also conducted in accordance with the package insert based on the concomitant use of antifungal drugs of CYP3A inhibitors. Drug reduction, withdrawal, and shortening of dosing days were allowed at the decision of the attending physician.

### Response criteria and assessments

Clinical responses were assessed using the modified International Working Group response criteria for AML [16]. Progressive disease (PD) and primary refractory disease was defined according to the recommendations of the European LeukemiaNet (ELN) 2017 [17]. Overall response rates (ORRs) included CR, CRi, morphologic leukemia-free state, and partial remission. Cytogenetic analysis was evaluated according to ELN 2017 [17]. OS was measured from the date of initial VEN administration to the date of death. EFS was measured from the date of initial VEN administration to the date of disease progression, confirmed relapse, or death of any causes.

### Measurement of *WT1* mRNA levels

Total RNA extraction was performed from white blood cells in PB. *WT1* mRNA was measured at SRL Co., Ltd. (Tokyo, Japan) or BML Co., Ltd. (Kawagoe city, Japan) by using the *WT1* mRNA assay kit II (Otsuka Pharmaceutical Co., Ltd., Tokyo, Japan) in accordance with the manufacturer’s instructions as previously described [18]. The lower limit of measurement for the *WT1* mRNA was 50 copies/µg RNA, and the patients were negative for *WT1* if the mRNA levels were below this limit. The *WT1* mRNA value in PB was assessed before and after each cycle of the VEN combination therapy.

### Statistical analysis

The clinical data cutoff date was September 30, 2022. Spearman's correlation coefficient was used to assess correlations between two ranked variables. All p-values were two-sided, and statistical significance was set at p ≤ 0.05. OS and EFS were estimated according to the Kaplan–Meier method. We used the log-rank test to compare survival curves between groups. To identify covariates that predict longer EFS, univariate and multivariate Cox regression analyses were performed. Multivariate models were analyzed including all the variables that had p-values < 0.10 in the univariate model. The HR between the groups was estimated with the Cox proportional hazards model with the same stratification factors. The 95% confidence intervals (CIs) were determined with the Clopper–Pearson exact method. Statistical analyses were performed using GraphPad Prism 9.4.1 (GraphPad Inc., San Diego, CA, USA).

## Results

### Patients and treatment

**Fig. 1:**
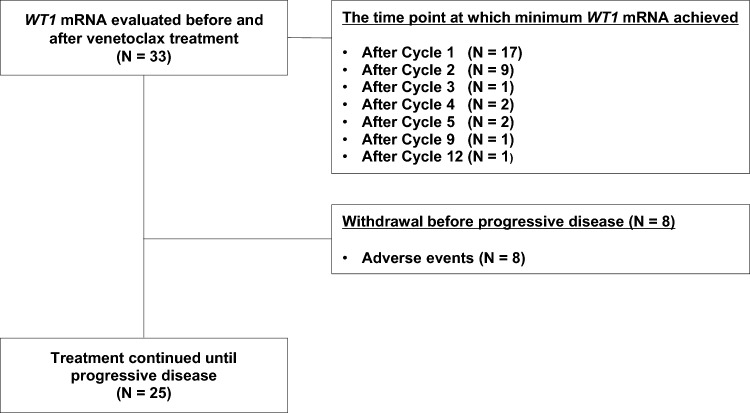
Flow diagram of patient disposition

From June 2021 to September 2022, 33 patients with untreated or relapsed/refractory AML who could be evaluated for PB WT1 dynamics in VEN combination therapy were analyzed in this study (Fig. 1).

Patient background is shown in Table 1. The median age was 73 years (range 39–87), and 45.0% of the patients were aged 75 years or older. Eastern Cooperative Oncology Group (ECOG) performance status (PS) scores were evaluated: PS 0–1 in 29 patients (88%) and PS 2–4 in 4 patients (12%). Fourteen patients were untreated AML and 19 patients were relapsed/refractory AML. Nineteen patients (58%) were classified as de novo AML and 14 patients (42%) as secondary AML. According to the French American British (FAB) classification, 25 patients (76%) were classified as M2 and 4 patients (12%) as M4/M5. Poor cytogenetic abnormalities were defined using the ELN 2017 [17]. Twelve (39%) of 31 patients tested were identified as having poor cytogenetic abnormalities such as 7 or 7q deletion, 5 or 5q deletion, and complex karyotype. The median *WT1* mRNA value before the VEN combination therapy was 3900 copies (range 190–77000). In the prior therapy group (n = 19), 13 patients (68%) had a history of AZA treatment.

**Table 1:** Baseline characteristics of the patients

Characteristics	All (n = 33)
Age, median (range)	73 (39–87)
< 75 years—no. (%)	18 (55)
≥ 75 years—no. (%)	15 (45)
Male sex, no. (%)	17 (52)
ECOG PS score, no. (%)	
0–1	29 (88)
2–4	4 (12)
AML status, no. (%)	
Untreated	14 (42)
Relapsed/Refractory	19 (58)
Prior history of relapse, no. (%)	
0	21 (64)
1	8 (24)
≥ 2	4 (12)
AML type, no. (%)	
De novo	19 (58)
Secondary	14 (42)
Secondary AML, no. (%)	
History of MDS	10 (71)
History of MPN	1 (7)
History of CMMoL	1 (7)
History of AA	1 (7)
Therapy-related AML	1 (7)
FAB classification, no./total no. (%)	
M0/M1	3/33 (9)
M2	25/33 (76)
M4/M5	4/33 (12)
Unclassified	1/33 (3)
Poor cytogenetic abnormalities^*1^, no./total no. (%)	12/31 (36)
Baseline WT1 value, median (range)	3900 (190–77000)
Prior treatment regimen, no./total no. (%)	
AZA	13/19 (68)
CAG or LDAC	7/19 (37)
DNR/Ara-C or IDA/Ara-C	7/19 (37)
HSCT	5/19 (26)

*ECOG* Eastern Cooperative Oncology Group, *PS* performance status, *MDS* myelodysplastic syndromes, *MPN* myeloproliferative neoplasms, *CMMoL* chronic myelomonocytic leukemia, *AA* aplastic anemia, *AML* acute myeloid leukemia, *FAB* French American British, *WT1* Wilms’ tumor – 1, *AZA* azacitidine, *CAG* low-dose cytarabine and aclarubicin in combination with granulocyte colony-stimulating factor, *LDAC* low-dose cytarabine, *DNR* daunorubicin, *Ara-C* cytarabine, *IDA* idarubicin, *HSCT* hematopoietic stem cell transplantation

^*1^Cytogenetic risk was evaluated according to the ELN 2017

Treatment exposure and tolerability are described in Table 2. AZA was selected as the combination drug with VEN in most cases (91%) and LDAC in only 3 patients (9%). Antifungal drugs were used in 79% of cases (fluconazole 88%, posaconazole 8%, voriconazole 4%). The median number of treatment cycles was 5 (range 1–14 cycles). The median observation period was 211 days (range 60–536 days). At the time of the cutoff date, 13 patients (39%) were still receiving the VEN combination therapy, and 20 (61%) had interrupted VEN combination therapy due to adverse events (AEs) (40%) and disease progression (60%).

**Table 2:** Treatment exposure and tolerability

Treatment characteristics	All (n = 33)
Combination drug with VEN, no (%)	
AZA	30 (91)
LDAC	3 (9)
Concomitant use of CYP3A inhibitor, no (%)	
None	7 (21)
Moderate CYP3A inhibitor (FLCZ)	23 (70)
Strong CYP3A inhibitor (PSCZ, VRCZ)	3 (9)
Total treatment cycles of VEN combination therapy, median (range)	5 (1–14)
Interruption of treatment at any cycles, no./total no. (%)	20/33 (61)
Due to adverse events	8/20 (40)
Due to disease progression	12/20 (60)

*VEN* venetoclax, *AZA* azacitidine, *LDAC* low-dose cytarabine, *FLCZ* fluconazole, *PSCZ* posaconazole, *VRCZ* voriconazole

### Response to VEN combination therapy

**Fig. 2:**
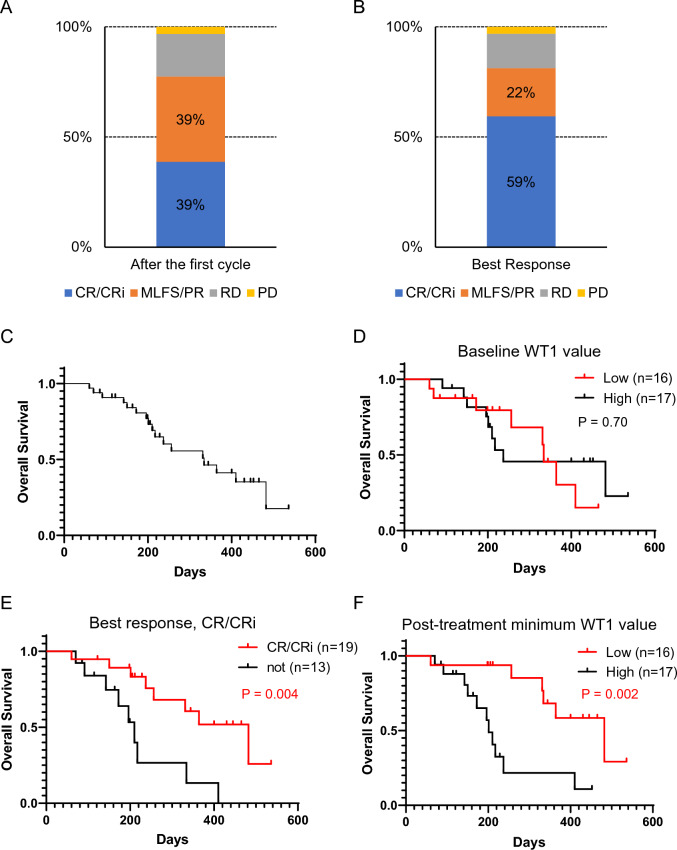
Treatment response and Kaplan–Meier curve of overall survival. The response rate after the first cycle (**A**) and the best response during the treatment course (**B**). **C**–**F** Kaplan–Meier curve of OS in all patients (**C**), baseline *WT1* mRNA value (median 3900 copy/μg RNA), low group (red) vs. high group (black) (**D**), best response, CR/CRi group (red) vs. no CR/CRi group (black) (**E**), and post-treatment minimum *WT1* mRNA value (median 690 copy/μg RNA), low group (red) vs. high group (black) (**F**). *CR/CRi* hematologic complete remission/complete remission with incomplete hematologic recovery, *MLFS/PR* morphologic leukemia-free state/partial remission, *RD* resistant disease, *PD* progressive disease

The treatment response and OS are shown in Fig. 2. The CR/CRi rates and ORRs after the first cycle were 39%, and 78%, respectively (Fig. 2A). The CR/CRi rates and ORRs as best response during the entire observation period were 59%, and 81%, respectively (Fig. 2B). The median OS was 334 days (95% CI 210–482) for all patients (Fig. 2C). No significant differences in OS were observed among patients stratified by baseline *WT1* mRNA value (median 3900 copy/μg RNA) (Fig. 2D).

In an analysis of post-treatment response, patients who achieved CR/CRi as the best response showed a significantly better OS than those who did not achieve CR/CRi (median OS 482 days (95% CI 237 to NA) and 210 days (95% CI 91–334), p = 0.004; Fig. 2E). With regard to stratification by post-treatment minimum *WT1* mRNA value at any time (Fig. 2F), which was divided into two groups with a median *WT1* mRNA value of 690 copy/μg RNA (n = 33), the low *WT1* mRNA group (n = 16) showed a significantly better OS than the high *WT1* mRNA group (n = 17) (median OS was 482 days (95% CI 331 to NA) and 201 days (95% CI 150–237, p = 0.002; Fig. 2F). The time points when the post-treatment minimum *WT1* mRNA value were achieved is shown in Fig. 1.

### Correlation between *WT1* mRNA levels in PB and the treatment response during the VEN combination therapy

**Fig. 3:**
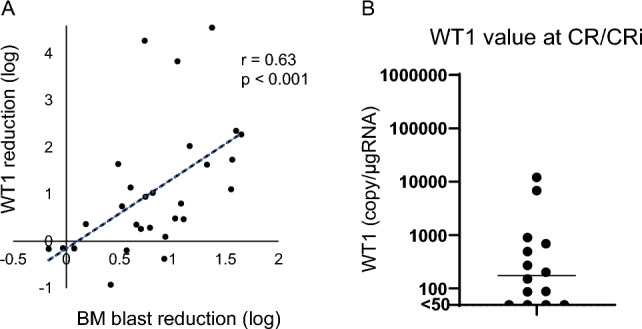
Correlation between *WT1* mRNA levels in peripheral blood and leukemia cells in the bone marrow. **A** Correlation between the reduction rate of blasts in the bone marrow (log reduction) and the reduction rate of *WT1* mRNA value in peripheral blood (log reduction). **B** Inter-individual variability of *WT1* mRNA value in peripheral blood among patients with CR/CRi. CR/CRi, hematologic complete remission/complete remission with incomplete hematologic recovery

When the bone marrow blast most decreased (n = 28), the correlation between the reduction rate of blasts in the bone marrow (log reduction) and the reduction rate of *WT1* mRNA value in PB (log reduction) was moderately correlated (r = 0.63; p < 0.001; Fig. 3A). However, the inter-individual variability of *WT1* mRNA value was found from negative to 12,000 (median 175 copy/μg RNA) at the time when patients achieved CR/CRi (Fig. 3B), suggesting the possibility of using *WT1* mRNA value as a marker for deeper remission than hematological CR.

### Reduction rate of *WT1* mRNA value by the end of cycle 2 is an independent prognostic factor for OS and EFS

**Fig. 4:**
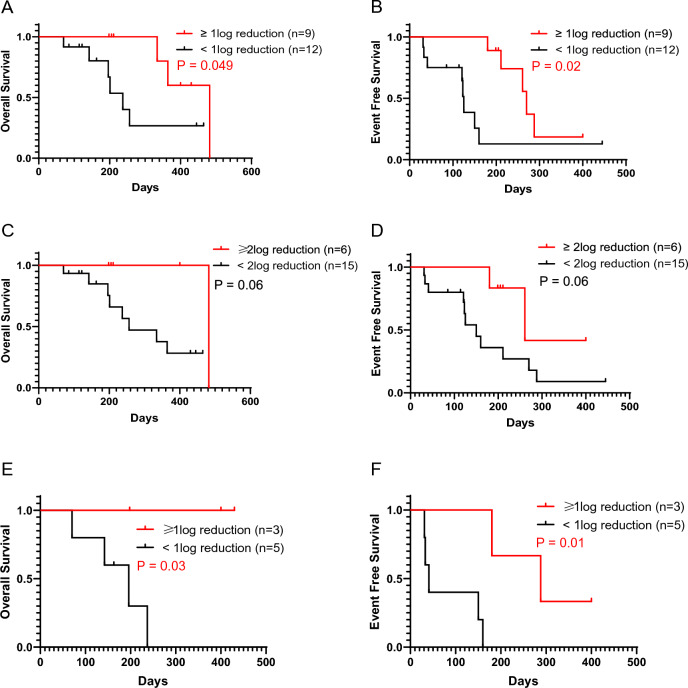
Prognostic impact of *WT1* mRNA reduction by the end of the second cycle. **A** Kaplan–Meier curve of overall survival for ≥ 1 − log reduction group (red; n = 9, median OS 482 days, 95% CI 334 to not available) and < 1 − log reduction group (black; n = 12, median OS 237 days, 95% CI 142 to not available). A significant difference was found between the two groups by the log-rank test (p = 0.049). **B** Kaplan–Meier curve of event-free survival for ≥ 1 − log reduction group (red; n = 9, median EFS 270 days, 95% CI 180 to not available) and < 1 − log reduction group (black; n = 12, median EFS 125 days, 95% CI 33–160). A significant difference was found between the two groups by using the log-rank test (p = 0.02). **C** Kaplan–Meier curve of overall survival for ≥ 2-log reduction group (red; n = 6, median OS 482 days, 95% CI not available to not available) vs. < 2-log reduction group (black; n = 15, median OS 256 days, 95% CI 196 to not available). No significant difference was found between the two groups by using the log-rank test (p = 0.06). **D** Kaplan–Meier curve of event-free survival for ≥ 2 − log reduction group (red; n = 6, median EFS 261 days, 95% CI 180 to not available) vs. < 2 − log reduction group (black; n = 15, median EFS 150, 95% CI 41 to 270). No significant difference was found between the two groups by using the log-rank test (p = 0.06). **E** Kaplan–Meier curve of overall survival for ≥ 1-log reduction group (red; n = 3, median OS not reached, 95% CI not available to not available) and < 1-log reduction group (black; n = 5, median OS 196 days, 95% CI 70 to not available) among patients with poor cytogenetic abnormalities. A significant difference was found between the two groups by the log-rank test (p = 0.03). **F** Kaplan–Meier curve of event-free survival for ≥ 1 − log reduction group (red; n = 3, median EFS 288 days, 95% CI 180 to not available) and < 1 − log reduction group (black; n = 5, median EFS 41 days, 95% CI 31 to not available) among patients with poor cytogenetic abnormalities. A significant difference was found between the two groups by the log-rank test (p = 0.01)

Of the 25 patients who could continue VEN combination therapy until PD (Fig. 1), the *WT1* mRNA value in PB was assessed in 21 patients by the end of cycle 2, and OS and EFS were stratified by a reduction rate of *WT1* mRNA value (log reduction) (Fig. 4). Patients with at least 1-log reduction in *WT1* mRNA values (n = 9) had significantly better OS and EFS compared with patients without 1-log reduction (n = 12) (median OS 482 days vs. 237 days, p = 0.049; median EFS 270 days vs. 125 days, p = 0.02) (Fig. 4A, B). Patients with at least 2-log reduction in *WT1* mRNA values (n = 6) showed better OS and EFS compared with patients with less than 2-log reduction (n = 15) (Fig. 4C, D). Furthermore, among patients with poor cytogenetic abnormalities and who could continue VEN combination therapy until PD, those with at least 1-log reduction in *WT1* mRNA values (n = 3) showed significantly better OS and EFS than those without 1-log reduction (n = 5) (median OS, NR vs. 196 days, p = 0.03; median EFS, 288 days vs. 41 days, p = 0.01) (Fig. 4E, F).

Univariate analysis showed that the presence of poor ECOG PS was a poor prognostic factor for EFS (HR 4.31, p = 0.07); however, this difference was not statistically significant. Shorter duration of the AZA + VEN treatment cycles was observed among the patients with poor PS (PS 2–4) group than among those in the better PS group (PS 0–1) (Median duration of treatment cycles, 3.5 vs. 6 cycles). The reason for treatment interruption was AEs (pneumonia and hematologic toxicity) in 50% of the patients in the poor PS group. Univariate analysis showed that at least 1-log reduction of *WT1* mRNA by the end of cycle 2 showed the good prognostic factor for EFS (HR 0.26, p = 0.024). Multivariate Cox proportional hazards regression analysis showed that at least 1-log reduction of *WT1* mRNA by the end of cycle 2 was an independent good prognostic factor for EFS (HR 0.26, 95% CI 0.078–0.84, p = 0.024; Table 3).

**Table 3:** Univariate and multivariate analyses for event-free survival (n = 25)

Variable	Univariate model			Multivariate model		
	HR	95% CI	p value	HR	95% CI	p value
Age, ≥ 75 years	0.70	0.24–2.06	0.52			
Sex, male	0.95	0.34–2.64	0.92			
ECOG PS, 2–4	4.31	0.89–20.1	0.07	2.02	0.37–11.02	0.42
AML type, de novo	1.00	0.35–2.85	1.00			
FAB classification, M4/M5	0.77	0.17–3.42	0.73			
Poor cytogenetic abnormalities^*1^	2.25	0.81–6.25	0.12			
Pre-treatment line, ≥ 1	1.84	0.63–5.38	0.27			
Number of previous relapse, ≥ 1	1.00	0.34–2.95	1.00			
Baseline LDH > ULN	1.96	0.69–5.55	0.21			
Best response by the end of the second cycle, CR/CRi	0.40	0.12–1.27	0.12			
WT1 reduction by the end of the second cycle, ≥ 1-log	0.26	0.078–0.84	0.024	0.26	0.078–0.84	0.024

*HR* hazard ratio, *CI* confidence interval, *ECOG* Eastern Cooperative Oncology Group, *PS* performance status, *FAB* French American British, *BM* bone marrow, *LDH* lactase dehydrogenase, *ULN* upper limit of normal, *CR* complete remission, *CRi* complete remission with incomplete hematologic recovery

^*^^1^Cytogenetic risk was evaluated according to the ELN 2017

### *WT1* mRNA negativity during the VEN combination therapy is an independent prognostic factor for OS and EFS

**Fig. 5:**
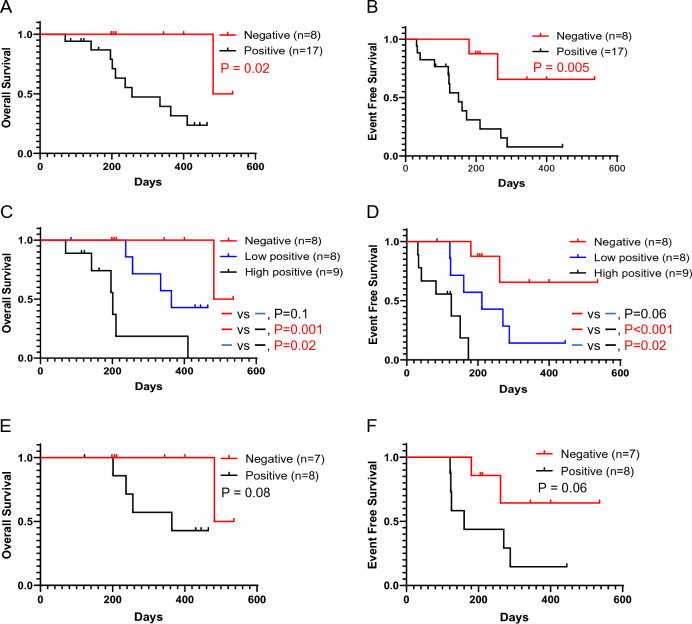
Prognostic impact of minimum *WT1* mRNA value during the VEN combination therapy. **A** Kaplan–Meier curve of overall survival for the *WT1* mRNA negative group (red; n = 8, median OS 482 days, 95% CI 482 to not available) vs. positive group (black; n = 17, median OS 256 days, 95% CI 196–410). A significant difference was found between the two groups by using the log-rank test (p = 0.02). **B** Kaplan–Meier curve of event-free survival for the *WT1* mRNA negative group (red; n = 8, median EFS not reached, 95% CI 180 to not available) vs. positive group (black; n = 17, median EFS 150 days, 95% CI–211). A significant difference was found between the two groups by using the log-rank test (p = 0.005). **C** Kaplan–Meier curve of overall survival for the *WT1* mRNA negative group (red; n = 8, median OS 482 days, 95% CI 482 to not available) vs. low positive group (blue, *WT1* mRNA value ≤ 700 copy/μg RNA; n = 8, median OS 364 days, 95% CI 237 to not available) vs. high positive group (black; *WT1* mRNA value > 700 copy/μg RNA; n = 9, median OS 201 days, 95% CI 70 to not available). A significant difference was found between the negative and high positive groups (p = 0.001) or low positive and high positive groups (p = 0.02) by using the log-rank test. **D** Kaplan–Meier curve of event-free survival for the *WT1* mRNA negative group (red; n = 8, median EFS not reached, 95% CI 180 to not available) vs. low positive group (blue, *WT1* mRNA value ≤ 700 copy/μg RNA; n = 8, median EFS 211 days, 95% CI 121–288) vs. high positive group (black; *WT1* mRNA value > 700 copy/μg RNA; n = 9, median EFS 125 days, 95% CI 31 to not available). A significant difference was found between the negative and high positive groups (p < 0.001) or low positive and high positive groups (p = 0.02) by using the log-rank test. **E** Kaplan–Meier curve of overall survival for the *WT1* mRNA negative group (red; n = 7; median OS, 482 days; 95% CI 482 to not available) vs. positive group (black; n = 8; median OS, 364 days; 95% CI 201 to not available) among patients with CR/CRi. No significant difference was found between the two groups using the log-rank test (p = 0.08). **F** Kaplan–Meier curve of event-free survival for the *WT1* mRNA negative group (red; n = 7, median EFS not reached, 95% CI 180 to not available) vs. positive group (black; n = 8; median EFS, 160 days; 95% CI, 121–288) among patients with CR/CRi. No significant difference was found between the two groups using the log-rank test (p = 0.06)

Of the 25 patients who could continue VEN combination therapy until PD (Fig. 1), the *WT1* mRNA value was evaluated during the clinical course at least once in all patients; OS and EFS were stratified by the value of post-treatment minimum *WT1* mRNA (Fig. 5). Patients with post-treatment *WT1* mRNA negative value (n = 8) showed significantly better OS and EFS than those without (n = 17) (median OS 482 days vs. 256 days, p = 0.02; median EFS NR vs. 150 days, p = 0.005) (Fig. 5A, B). Eight of the 25 patients achieved post-treatment *WT1* mRNA negative value: four patients by the end of cycle 1, two patients by the end of cycle 4, and two patients by the end of cycle 10. When patients with post-treatment *WT1* mRNA positive value (n = 17) were divided into two groups by a median *WT1* mRNA value, OS and EFS were significantly better in the post-treatment *WT1* mRNA negative group (n = 8) and low positive group (n = 8) compared with the high positive group (n = 9) (median OS 482 days vs. 364 days vs. 201 days, median EFS NR vs. 211 days vs. 125 days) (Fig. 5C, D). In NPM1-mutated AML cases, the measurement of NPM1 levels is generally recommended as an MRD marker. As for the usefulness of WT1 monitoring as an MRD marker in genetic variant cases, we investigated a case of NPM1-mutated AML enrolled in the present study with regard to the usefulness of WT1 monitoring as an MRD marker instead of the measurement of the NPM1 levels. A 77-year-old woman with NPM1-mutated AML achieved CR after the third cycle of VEN/AZA therapy, and WT1 negativity was confirmed after the fifth cycle of VEN/AZA therapy with a 4-log reduction in its levels compared with the pre-treatment WT1 value. The patient has been continuing the VEN/AZA therapy for 536 days without any events. Therefore, WT1 monitoring could serve as an MRD marker, even in patients with some genetic variants. Furthermore, among patients who showed the best response to CR/CRi, those with post-treatment *WT1* mRNA negative values (n = 7) showed a trend toward better OS and EFS than those without post-treatment *WT1* mRNA negative values (n = 8) (median OS, 482 days vs. 364 days, p = 0.08; median EFS, NR vs. 160 days, p = 0.06) (Fig. 5E, F). Multivariate Cox proportional hazards regression analysis showed that a negative *WT1* mRNA value during the clinical course was an independent prognostic factor for EFS (HR 0.15, 95% CI 0.033–0.67; P = 0.013).

### Correlation between *WT1* mRNA dynamics during the VEN combination therapy and OS and EFS

**Fig. 6:**
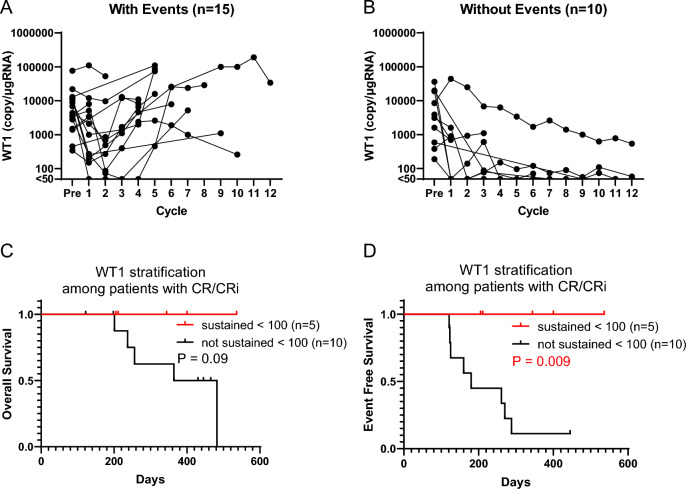
Prognostic impact of *WT1* mRNA dynamics. **A**, **B** Dynamics of *WT1* mRNA values in patients with or without any events. **C**, **D** The overall survival and event-free survival stratified by the dynamics of *WT1* mRNA values after treatment among patients with CR/CRi (n = 15). **C** Kaplan–Meier curve of overall survival for *WT1* mRNA sustained < 100 group (red; n = 5, median OS not reached, 95% CI not available to not available) and not sustained < 100 group (black; n = 10, median OS 423 days, 95% CI 201 to not available). No significant difference was found between the two groups by using the log-rank test (p = 0.09). **D** Kaplan–Meier curve of event-free survival for *WT1* mRNA sustained < 100 group (red; n = 5, median EFS not reached, 95% CI not available to not available) and not sustained < 100 group (black; n = 10, median EFS 180 days, 95% CI 121–288). A significant difference as found between the two groups by using the log-rank test (p = 0.009). CR/CRi, hematologic complete remission/complete remission with incomplete hematologic recovery

Figure 6A and B show the dynamics of *WT1* mRNA values in PB during the clinical course in patients who could continue VEN combination therapy until PD (n = 15 in the group with events and n = 10 in the group without events). The event details are as follows: disease progression (n = 4), recurrence (n = 8), and death of any causes (n = 3). Among the patients without any events during the clinical course, the *WT1* mRNA values in PB showed a declining trend. On the other hand, among the patients with any events during the clinical course, the *WT1* mRNA values in PB showed a sustained increase or re-increase after a decrease. An increase in the WT1 value preceding any events (progressive disease in 3 patients and relapse in 5 patients) was confirmed in at least 8 of the 15 patients, as shown in Fig. 6A. The median number of days from the increase in WT1 expression to event occurrence was 49 days (range, 20–142). In the other seven cases, the association between WT1 level increase and event occurrence could not be evaluated correctly because WT1 level measurement and treatment response were not evaluated in every cycle. Patients without events showed significantly better OS and EFS than those with events (median OS NR vs. 256 days, p = 0.01, median EFS NR vs. 150 days, p < 0.001). Of the 25 patients who could continue VEN combination therapy until PD, 15 patients achieved CR/CRi. Among these 15 patients who achieved CR/CRi, we evaluated the correlation between the dynamics of *WT1* mRNA in PB and OS and EFS. As a result, patients in whom *WT1* mRNA values decreased to < 100 copy/μg RNA and then sustained < 100 (n = 5) showed better OS and significantly better EFS than in those without (n = 10) (median OS NR vs. 423 days, p = 0.09, median EFS NR vs. 180 days, p = 0.009) (Fig. 6C, D). Therefore, even in patients who achieved hematologic CR, the dynamics for *WT1* mRNA values in PB could be an MRD marker for long-term prognosis.

## Discussion

In this study, we showed the prognostic impact of post-treatment *WT1* mRNA dynamics in 33 patients with untreated or relapsed/refractory AML treated with VEN combination therapy.

The CR/CRi rate was 39% after cycle 1, and 59% during the entire observation period. The median OS was 334 days (95% CI 210 to 482). In the phase 3 trial of the VIALE-A study for patients with AML ineligible for intensive chemotherapy, the median OS was 14.7 months (~ 448 days) in the VEN/AZA group and the CR/CRi rate was 43.4% after cycle 1 and 66.4% during the study period [4]. Thus, CR/CRi rates in our cohort were comparable to the VIALE-A study.

MRD assessment is important for predicting treatment response and relapse in AML [8]. The two most commonly used and recommended techniques for MRD monitoring are MFC and real-time quantitative PCR (RT-PCR)-based methods [19]. However, the standardized technique of MFC, which requires bone marrow samples, has not been fully developed in clinical practice, and the sensitivity of MRD detection is insufficient (10^–3^). By contrast, RT-PCR methods are very sensitive, rapid, and well standardized. However, RT-PCR techniques are applicable only for patients who have a suitable target gene such as the chimeric gene fusions [12]. In this regard, *WT1* mRNA overexpression is observed at least 80% of the patients with AML, that is, *WT1* mRNA could be a universal MRD marker even in patients with AML without disease-specific fusion genes [11, 12]. In our cohort, baseline *WT1* mRNA overexpression was observed in 87.5% (42/48) of the patients.

The number of leukemic cells in the bone marrow is known to correlate with *WT1* mRNA levels in the bone marrow [20, 21], and a significant decrease in *WT1* mRNA levels in PB during treatment is correlated with good blast clearance [22]. Furthermore, several reports have revealed that the expression level of *WT1* mRNA and the data of MFC and RT-PCR are consistent and equally predict clinical outcome [23]. Although *WT1* mRNA values could be evaluated in both bone marrow and PB, bone marrow samples could be influenced by the background level of WT1 gene expression. Therefore, *WT1* mRNA evaluation in PB, which could be evaluated more simply than bone marrow, could more accurately predict relapse [24]. In this study, we found a positive correlation between a reduction rate of bone marrow leukemic cells (log reduction) and a reduction rate of *WT1* mRNA values in PB (log reduction). Therefore, *WT1* mRNA evaluation in PB is a universal surrogate marker of leukemic persistence. Monitoring the *WT1* mRNA levels in PB may be useful, especially for patients with long-lasting myelosuppression in MLFS cases and for those in whom frequent bone marrow examination is difficult.

Several studies have shown that a 1-log reduction in *WT1* mRNA value in PB is a predictor of relapse-free survival and a 2-log or greater reduction in *WT1* mRNA value is associated with significantly better OS [13–15]. In addition, *WT1* mRNA value positivity (≥ 50 copies/µg RNA) in PB after intensive chemotherapy or stem cell transplantation is an independent poor prognostic factor for relapse [24–28]. Therefore, *WT1* mRNA value after chemotherapy can indicate disease activities such as remission and relapse [29]. This study also found that *WT1* mRNA 1-log reduction by the end of cycle 2 and *WT1* mRNA value negativity during the treatment cycle were associated with significantly prolonged OS and EFS. The *WT1* mRNA 1-log reduction by the end of cycle 2 significantly prolonged OS and EFS also in patients with poor cytogenetic abnormalities.

We also found that the dynamics of *WT1* mRNA value in PB could predict clinical outcome, that is, no increase in *WT1* mRNA was associated with better EFS and an increase in *WT1* mRNA was associated with relapse. Furthermore, *WT1* mRNA values at the time of CR/CRi achievement varied from negative to 12,000 (median 175 copies/μg RNA), and *WT1* mRNA was expected to be a marker of MRD even in patients who achieved hematologic CR. Among patients who achieved CR/CRi (n = 15), post-treatment *WT1* mRNA negativity (n = 7) showed a trend toward better OS and EFS than for those without (n = 8) (median OS 482 days vs. 364 days, p = 0.08; median EFS NR vs. 160 days, p = 0.06). Although the log-rank test did not show a statistically significant difference, the small number of patients may preclude accurate statistical analysis. Among patients who achieved CR/CRi (n = 15), the post-treatment *WT1* mRNA values to be sustained < 100 copies/μg RNA (n = 5) showed significantly better EFS (median EFS NR vs. 180 days, p = 0.009). These results were obtained in patients with AML treated with VEN combination therapy until PD.

In this study, WT1 mRNA expression was quantified as the ratio of WT1 mRNA levels to GAPDH mRNA levels, and which is not a universally validated MRD assay method. In this regard, the measurement of WT1 levels in this study did not use the ABL1 adjustment; thus, it cannot be compared with that in a previously reported paper [24–28].

This study has several limitations. First, the number of sample sizes was small, and the duration of the observation period was short. Second, treatment adjustment at the decision of the attending physician was allowed. Finally, the risk category of the ELN classification could not be applied because NPM1 and FLT3 mutations were not evaluated in all patients. Therefore, increased number of patients and long-term follow-up are warranted in future investigations.

## Conclusion

To the best of our knowledge, this study is the first study to describe the prognostic value of *WT1* mRNA in PB as an MRD marker in patients with AML treated with VEN combination therapy. In conclusion, monitoring of *WT1* mRNA, which can be easily evaluated through PB sampling, could be a universal MRD marker for almost all AML patients treated with VEN combination therapy to predict disease prognosis.

## Declarations

### Statements and declarations

The multicenter prospective observational study was conducted in Akita prefecture, Japan. This study enrolled patients with AML who were judged ineligible for intensive chemotherapy by the attending physician and treated by VEN combination therapy. It was conducted in accordance with the 1964 Helsinki Declaration and approved by the Ethics Committee of Akita University (August 6, 2021/No. 2696). Written informed consent was obtained from all participants before enrollment.

## References

[1] Appelbaum FR, Gundacker H, Head DR (2006). Age and acute myeloid leukemia. Blood.

[2] Medeiros BC, Satram-Hoang S, Hurst D (2015). Big data analysis of treatment patterns and outcomes among elderly acute myeloid leukemia patients in the United States. Ann Hematol.

[3] Shallis RM, Wang R, Davidoff A (2019). Epidemiology of acute myeloid leukemia: recent progress and enduring challenges. Blood Rev.

[4] DiNardo CD, Jonas BA, Pullarkat V (2020). Azacitidine and venetoclax in previously untreated acute myeloid leukemia. N Engl J Med.

[5] Wei AH, Montesinos P, Ivanov V (2020). Venetoclax plus LDAC for newly diagnosed AML ineligible for intensive chemotherapy: a phase 3 randomized placebo-controlled trial. Blood.

[6] Döhner H, Weisdorf DJ, Bloomfield CD (2015). Acute myeloid leukemia. N Engl J Med.

[7] Ravandi F, Walter RB, Freeman SD (2018). Evaluating measurable residual disease in acute myeloid leukemia. Blood Adv.

[8] Heuser M, Freeman SD, Ossenkoppele GJ (2021). 2021 Update on MRD in acute myeloid leukemia: a consensus document from the European LeukemiaNet MRD Working Party. Blood.

[9] Pratz KW, Jonas BA, Pullarkat V (2022). Measurable residual disease response and prognosis in treatment-naïve acute myeloid leukemia with venetoclax and azacitidine. J Clin Oncol.

[10] Gessler M, Poustka A, Cavenee W (1990). Homozygous deletion in Wilms tumours of a zinc-finger gene identified by chromosome jumping. Nature.

[11] Cilloni D, Gottardi E, De Micheli D (2002). Quantitative assessment of WT1 expression by real time quantitative PCR may be a useful tool for monitoring minimal residual disease in acute leukemia patients. Leukemia.

[12] Lazzarotto D, Candoni A (2022). The role of Wilms' tumor gene (WT1) expression as a marker of minimal residual disease in acute myeloid leukemia. J Clin Med.

[13] Gianfaldoni G, Mannelli F, Ponziani V (2010). Early reduction of WT1 transcripts during induction chemotherapy predicts for longer disease free and overall survival in acute myeloid leukemia. Haematologica.

[14] Gray JX, McMillen L, Mollee P (2012). WT1 expression as a marker of minimal residual disease predicts outcome in acute myeloid leukemia when measured post-consolidation. Leuk Res.

[15] Šálek C, Vydra J, Cerovská E (2020). WT1 expression in peripheral blood at diagnosis and during the course of early consolidation treatment correlates with survival in patients with intermediate and poor-risk acute myeloid leukemia. Clin Lymphoma Myeloma Leuk.

[16] Cheson BD, Bennett JM, Kopecky KJ (2003). Revised recommendations of the International Working Group for diagnosis, standardization of response criteria, treatment outcomes, and reporting standards for therapeutic trials in acute myeloid leukemia. J Clin Oncol.

[17] Döhner H, Estey E, Grimwade D (2017). Diagnosis and management of AML in adults: 2017 ELN recommendations from an international expert panel. Blood.

[18] Miyawaki S, Hatsumi N, Tamaki T (2010). Prognostic potential of detection of WT1 mRNA level in peripheral blood in adult acute myeloid leukemia. Leuk Lymphoma.

[19] Ommen HB (2016). Monitoring minimal residual disease in acute myeloid leukaemia: a review of the current evolving strategies. Ther Adv Hematol.

[20] Alonso-Dominguez JM, Tenorio M, Velasco D (2012). Correlation of WT1 expression with the burden of total and residual leukemic blasts in bone marrow samples of acute myeloid leukemia patients. Cancer Genet.

[21] Giudice V, Gorrese M, Vitolo R (2021). WT1 expression levels combined with flow cytometry blast counts for risk stratification of acute myeloid leukemia and myelodysplastic syndromes. Biomedicines.

[22] Kern W, Haferlach T, Schoch C (2003). Early blast clearance by remission induction therapy is a major independent prognostic factor for both achievement of complete remission and long-term outcome in acute myeloid leukemia: data from the German AML Cooperative Group (AMLCG) 1992 Trial. Blood.

[23] Polak J, Hajkova H, Haskovec C (2013). Quantitative monitoring of WT1 expression in peripheral blood before and after allogeneic stem cell transplantation for acute myeloid leukemia - a useful tool for early detection of minimal residual disease. Neoplasma.

[24] Duléry R, Nibourel O, Gauthier J (2017). Impact of Wilms' tumor 1 expression on outcome of patients undergoing allogeneic stem cell transplantation for AML. Bone Marrow Transplant.

[25] Weisser M, Kern W, Rauhut S (2005). Prognostic impact of RT-PCR-based quantification of WT1 gene expression during MRD monitoring of acute myeloid leukemia. Leukemia.

[26] Cilloni D, Renneville A, Hermitte F (2009). Real-time quantitative polymerase chain reaction detection of minimal residual disease by standardized WT1 assay to enhance risk stratification in acute myeloid leukemia: a European LeukemiaNet study. J Clin Oncol.

[27] Lambert J, Thomas X, Marceau-Renaut A (2021). Early detection of WT1 measurable residual disease identifies high-risk patients, independent of transplantation in AML. Blood Adv.

[28] Rautenberg C, Lauseker M, Kaivers J (2021). Prognostic impact of pretransplant measurable residual disease assessed by peripheral blood WT1-mRNA expression in patients with AML and MDS. Eur J Haematol.

[29] Liu H, Wang X, Zhang H (2019). Dynamic changes in the level of WT1 as an MRD marker to predict the therapeutic outcome of patients with AML with and without allogeneic stem cell transplantation. Mol Med Rep.

